# Legionnaires Disease Associated with a Private-Use Hot Tub in a Vacation Rental Property — New York, October 2024–April 2025

**DOI:** 10.15585/mmwr.mm7522a1

**Published:** 2026-06-11

**Authors:** Matthew Morse, Martin Zartarian, Braden Savage, Patrick O’Connor, David Nicholas, Halex Jones, Christopher Gil, Danielle Wroblewski, Stacey Chmura, Nirupam Biswas, Craig Bocketti, Grace Willard, Seth Blumerman, Theresa Hattenrath, Donna Gowie, Lisa Mingle, Ursula Lauper

**Affiliations:** ^1^New York State Department of Health, Bureau of Water Supply Protection; ^2^New York State Department of Health, Bureau of Community Environmental Health and Food Protection; ^3^Department of Epidemiology & Biostatistics, College of Integrated Health Sciences, University at Albany, State University of New York, Albany, New York; ^4^New York State Department of Health, Bureau of Communicable Disease Control; ^5^New York State Department of Health, Wadsworth Center, Bacteriology Laboratory; ^6^New York State Department of Health, Wadsworth Center, Division of Environmental Health Sciences.

**Summary**

**What is already known about this topic?**

Legionnaires disease results from inhalation of aerosolized *Legionella* bacteria from contaminated water. Hot tubs create aerosols and maintain water temperatures within a favorable range for *Legionella* growth. Private short-term rental properties are not subject to the same public health regulations as commercial properties.

**What is added by this report?**

A 2024 New York Legionnaires disease investigation used whole genome sequencing to identify a rental property hot tub as the source of two cases of Legionnaires disease. Health department officials used a public nuisance law to ensure that the hot tub was properly remediated before reopening.

**What are the implications for public health practice?**

Efforts to raise awareness among vacation rental and other short-term rental property owners of the risks associated with improperly managed hot tubs could reduce the risk for Legionnaires disease. Travelers staying in vacation rental properties should be aware that use of hot tubs might pose a risk for Legionnaires disease, particularly those travelers with underlying medical conditions.

## Abstract

In mid-October 2024, the New York State Department of Health (NYSDOH) was notified of two cases of Legionnaires disease in persons who stayed at the same short-term vacation rental property and had both used the rental property’s hot tub. The diagnoses were confirmed by urine antigen tests, and NYSDOH successfully isolated *Legionella pneumophila* serogroup 1 from the sputum of one patient. Local health department staff members collected three samples from three sinks and two samples from two showers to assess the rental property’s potable water system, which was supplied by a private well. Three samples were also collected from the rental property’s hot tub. Whole genome sequencing of isolates from the hot tub samples and the sputum specimen were closely related, suggesting that the hot tub was the likely source of exposure. Hot tubs create aerosols and typically maintain water temperatures of approximately 100°F–104°F (38°C–40°C). This temperature is within the most favorable range for *Legionella* growth and also accelerates the decay of disinfectants. Guidance from NYSDOH and CDC regarding proper operation, disinfection, and remediation of hot tubs was provided to the rental property owner. NYSDOH recommended that the owner close the hot tub until proper remediation was performed and postremediation samples without detection of any *Legionella* bacteria were collected by NYSDOH staff members. The rental property owner did not initially comply with NYSDOH recommendations, and a public nuisance law was used to ensure that proper measures were taken to disinfect the hot tub before use by future guests. Activities to raise awareness among short-term rental and vacation rental property owners regarding the risks associated with an improperly managed hot tub are needed to decrease the likelihood that rental property hot tubs are in a condition that is conducive to *Legionella* growth and reduce the risk for Legionnaires disease associated with vacation rental stays. Travelers staying in vacation rental properties should be aware that use of hot tubs might pose a risk for Legionnaires disease and exercise caution when using hot tubs, particularly those travelers with underlying medical conditions.

## Introduction

In mid-October 2024, the New York State Department of Health (NYSDOH) was notified of an increase in in the number of reported cases incidence of Legionnaires disease in a western New York county (county A). Five patients in the same city (city A) had received positive urine antigen test (UAT) results, indicating recent exposure to *Legionella* *pneumophila* serogroup 1 (during 2022–2024, county A averaged 22 cases per year. Two of these patients were family members who had spent several nights together at a short-term vacation rental property in city B, 40 miles south of city A. NYSDOH began an investigation of the rental property in an effort to ascertain the extent of this outbreak, identify any additional cases, and investigate any potential sources of exposures so that proper remedial and preventive action could be taken.

## Investigations and Results

### Epidemiologic Investigation

**Initial investigation.** Epidemiology staff members from the local health department interviewed five persons (patients A, B, C, D, and E) who received positive UAT results using the NYSDOH Legionnaires Disease Questionnaire (part of the standard case report form developed based on CDC’s *Legionella* case report form) (Table 1). Two family members (patients A and B) who had stayed together at the rental property and had both used the hot tub, sauna, and shower reported similar symptom onset dates during the first week of October 2024. This outbreak investigation work represents standard public health practice by NYSDOH, and institutional review board review was not required.

**TABLE 1:** Age, month of symptom onset, exposure history, and test results among five persons with Legionnaires disease,* including two who used a hot tub at the same vacation rental property — New York, 2024

Patient	Part of rental property investigation^†^	Age, yrs	Month of symptom onset	Sputum *Legionella* species culture result^§^	Exposure history	Test results
A^¶^	Yes	68	Oct	*Legionella pneumophila* serogroup 1 isolated**	Use of hot tub and sauna; shower at rental property	WGS from clinical sample closely related to environmental samples from rental property hot tub
B	Yes	33	Oct	No sputum sample collected	Use of hot tub and sauna; shower at rental property	No clinical sample collected, but environmental and epidemiologic evidence suggest infection caused by rental property hot tub exposure
C	No	48	Sep	Negative	Use of private residential hot tub and possible occupational exposure	Possible occupational exposure investigated; no exposure source confirmed
D	No	94	Oct	No sputum sample collected	Outpatient medical appointments and local restaurant visits	Patient unable to produce sputum; exposure source not identified
E	No	72	Sep	*L. pneumophila* isolated	Outpatient medical appointment visits, single-night stay at local hotel, and use of private residential hot tub	No match between WGS results from clinical sample and anything in the database; exposure source not identified

**Abbreviation:** WGS = whole genome sequencing.

* Confirmed by urine antigen testing.

^†^ Although the initial cluster notification included five confirmed cases in persons living within the same county, interviews identified common exposures of patients A and B at the rental property in a neighboring county, leading to a separate outbreak investigation. Interviews also revealed that five additional guests stayed at the rental property and might have used the hot tub. These additional guests did not report any symptoms and therefore were not interviewed or part of any investigative process. Patients C, D, and E were investigated as a separate cluster. No additional exposures sources were identified.

^§^ For a sputum sample to be collected and analyzed, the sample must be ordered by a physician, the ill persons must be able to produce sputum, and *Legionella* organisms must be isolated from the sample via culture before the species can be sequenced. Administering antibiotics before collecting the sample might reduce the likelihood of isolating *Legionella* species from a sputum sample.

^¶^ Patient A had preexisting hypertension and cardiovascular disease, was hospitalized, and received antibiotics and invasive mechanical ventilation. The patient survived, but the hospital discharge date is not known.

** Test results from a commercial laboratory for a sputum sample from patient A initially were negative, but *Legionella pneumophila* serogroup 1 was isolated by culture by the New York State Department of Health public health laboratory by culture.

**Investigation of the rental property (patients A and B).** After identification of the common exposures of patients A and B, and because Legionnaires disease has been associated with hot tub use (*1*,*2*), an investigation of the rental property was conducted. A confirmed case of Legionnaires disease associated with the rental property was defined as receipt of a positive UAT or *Legionella* species culture result in a person with a clinically compatible illness who reported a stay at the rental property in October 2024. Patient A and patient B experienced cough, fever, and chills. Patient A, who had comorbid conditions, was hospitalized and received antibiotics and invasive mechanical ventilation (Clinical Guidance for Legionella Infections | CDC) (Table 1). Patient A survived but the hospital discharge date is not known. Patient B visited an emergency department and was prescribed antibiotics but was not admitted. The results from a sputum sample collected from patient A were initially negative at a commercial laboratory. NYSDOH requested that the specimen be forwarded to the Wadsworth Center David Axelrod Institute (WC-DAI), a public health laboratory operated by NYSDOH, for further testing. No clinical specimen was collected from patient B. A line list of patients with Legionnaires disease associated with the rental property was developed by NYSDOH, which included demographic characteristics, signs and symptoms, underlying medical conditions, symptom onset date, travel and exposure history, and diagnostic testing results. Retrospective and prospective reviews of case exposure histories did not identify any additional cases associated with the rental property.*

**Investigation of patients C, D, and E.** A separate cluster investigation was conducted for patients C, D, and E; no common exposures were identified among these patients (Table 1). A potential occupational exposure was investigated for patient C, but no source was identified.

### Rental Property Environmental Health and Laboratory Investigation

**Collection of samples.** Eight samples were collected from the rental property (Table 2): five potable water samples from the sinks and showers, two swabs of hot tub surfaces, and one nonpotable water sample collected directly from the hot tub basin. Each water sample was initially collected into a sterile 34-oz (1-L) bottle and subsequently divided into 4-oz (120-mL) and 10-oz (290-mL) sterile plastic bottles containing sodium thiosulfate, which was used to neutralize chlorine in the sample and prevent further disinfection during transport to the laboratory and to ensure preservation of any viable *Legionella *species present in the sample.^†^

**TABLE 2:** Location, source type, and test results for samples collected from a vacation rental property associated with two cases of Legionnaires disease — New York, 2024

Sample collection location	Source type	Legiolert (MPN/mL)*	Culture (CFU/mL)^†^	WGS (SNP difference)^§^
Kitchen bathroom sink (cold water)	Potable water	<0.01	Not detected	NA
Primary bathroom shower (first draw, hot water)	Potable water	<0.01	Not detected	NA
Primary bathroom sink (first draw, hot water)	Potable water	<0.01	Not detected	NA
Basement shower (first draw, hot water)	Potable water	<0.01	Not detected	NA
Basement sink (first draw, hot water)	Potable water	<0.01	Not detected	NA
Hot tub water	Nonpotable water	134.2	Not detected	2
Hot tub swab (air-water interface)	Nonpotable water, swab	13,677	3,700	2
Hot tub swab (filter)	Nonpotable water, swab	13,677	Not detected	3

**Abbreviations**: CFU = colony-forming unit; MPN = most probable number; NA = not applicable; SNP = single nucleotide polymorphism; WGS = whole genome sequencing.

* This culture-based method only detects *Legionella pneumophila*; MPN represents the most probable number of colonies of *L. pneumophila* detected per 1 mL of a sample. A liquid-based enzyme substrate is incubated with a sample; after 7 days, the number of positive wells is counted, and an MPN table is used to determine the concentration *of L. pneumophila* (MPN/mL) in the original sample.

^†^ The number of CFUs of *Legionella *species detected per 1 mL of a sample. Potable water samples were invalid because free chlorine exceeded 10 mg/L.

^§^ The number of SNPs compared with the patient sample obtained from investigation.

**Analysis of water samples.** All eight collected water samples were submitted to the Wadsworth Center Environmental Biggs Laboratory for analysis both by IDEXX’s Legiolert and by standard culture (International Organization for Standardization [ISO] 11731:2017) (*3*). Legiolert is a culture-based method that can be conducted more quickly (7 days) than standard culture (10–14 days).^§^ Use of both methods increases the chances of recovering an organism and increases the likelihood of detecting any *Legionella* species in the sample.

**Analysis of swab samples.** Because the Legiolert test is only intended for use with water samples, the laboratory created a modified protocol for use with swabs. Swabs were prepared according to ISO 11731:2017 (*3*), generating 1:10 and 1:100 serial dilutions in phosphate-buffered saline. A 0.03-oz (1-mL) aliquot of each dilution was added to 3 oz (99 mL) of reagent water (highly purified water with no detectable concentration of the compound or element to be analyzed) and processed according to the manufacturer’s instructions for potable water.

**Identification of *L. pneumophila* from hot tub samples.** Legiolert detected *L. pneumophila* in all three hot tub samples, ranging from 134 to 13,677 most probable number (MPN) of colonies per milliliter^¶ ^(Table 2). Legiolert did not detect *L. pneumophila* in the potable water system samples. Standard culture (ISO 11731:2017) confirmed the presence of *Legionella* species in one sample, the swab from the air-water interface of the hot tub (3,700 colony-forming units per milliliter). The potable water samples were found to contain a total and free chlorine residual >10 mg/L, and the sodium thiosulfate contained in the sterile plastic bottles used for sample collection can neutralize up to 15 mg/L of chlorine, suggesting the presence of >25 mg/L. The high residual chlorine level would normally result in rejection of a sample, but the laboratory agreed to analyze these samples at the request of NYSDOH even though isolation of viable *Legionella* organisms from the potable water samples was deemed highly unlikely.

**Whole genome sequencing (WGS) of isolates.** WC-DAI performed the analysis and WGS of clinical specimens and environmental samples. A sputum sample from patient A that initially tested negative by culture at a commercial laboratory was forwarded to WC-DAI, which isolated *L.* *pneumophila* serogroup 1 via culture. The isolates from the hot tub differed by two to three single nucleotide polymorphisms from patient A’s clinical isolate, indicating that they were closely related.

## Public Health Response

### Recommendations to the Rental Property Owner

Discussion between NYSDOH and the property manager revealed that the owner had disinfected the private well with a large amount of liquid chlorine bleach the night before health department officials were scheduled to visit the site. NYSDOH staff members learned that the hot tub was not regularly disinfected and recommended the hot tub immediately be closed to guests until it was cleaned professionally, and a chemical treatment program was implemented. CDC guidance was provided to the property owner (*4*), who was instructed to notify NYSDOH when proper remediation of the hot tub was completed.

### Follow-Up After Remediation Recommendations

Approximately 4 weeks after sample collection and recommendations were provided to the rental property owner, local health department officials informed NYSDOH that the hot tub was back in use; NYSDOH had not been notified by the property owner. The rental property owner had personally cleaned the hot tub (i.e., did not hire professionals), tested a sample using an unapproved method, and reopened the hot tub for guest use without consulting NYSDOH. NYSDOH staff members reiterated the importance of having the equipment cleaned professionally and allowing NYSDOH staff members to collect the samples for analysis by a laboratory certified by the New York Environmental Laboratory Approval Program (*5*). After an additional 4 weeks, NYSDOH staff members were notified that the rental listing still advertised the hot tub, and guests were leaving new reviews that mentioned hot tub use.

### Use of the New York Public Nuisance Law

Because the hot tub had not been satisfactorily disinfected, and the rental property owner continued to allow its use by guests, local health department officials charged the owner with violation of the Erie County Sanitary Code, and the hot tub was deemed a public nuisance. The health department also issued a commissioner’s order to close the hot tub until it was deemed safe to use.

### Subsequent Remediation and Testing Results

After the hot tub was cleaned and disinfected by a professional, two rounds of follow-up sampling were conducted by the local health department staff members. The commissioner’s order was lifted on March 31, 2025, after two successive rounds of sampling from the hot tub did not identify any viable *Legionella* organisms. The professional hired by the rental property to clean the hot tub was subsequently hired to service it weekly.

## Discussion

By positively matching an environmental source of *L.* *pneumophila *with Legionnaires disease cases (*6*), WGS determined that the clinical specimen collected from patient A was closely related to the environmental samples collected from the hot tub, thereby establishing it as likely source of infection. Rental property hot tub use is frequently associated with reports of Legionnaires disease, and NYSDOH has successfully matched clinical samples from persons with Legionnaires disease to environmental samples from hot tubs in recent years (*1*,*6*).

According to CDC’s Supplementary Legionnaires Disease Surveillance System, during 2014–2021, one in seven patients with Legionnaires disease report having stayed overnight in a hotel or vacation rental property; among those, approximately one half report having used a hot tub (*1*). A 2021 NYSDOH investigation concluded that at least one case of Legionnaires disease was associated with shared use of a private property hot tub (*6*), and CDC recently reported that two Legionnaires disease outbreaks were associated with outdoor hot tubs intended for private use on cruise ships (*2*). NYSDOH staff members added guidance to the program website designed to raise awareness about the risks involved with the use of an improperly managed hot tub and included guidance for proper routine water treatment practices.** New York has regulations designed to prevent *Legionella* contamination in cooling towers and in certain health care facilities^††^ (*7*) and to ensure that proper sanitary practices are followed in public pools (*8*); however, residential spa pools, including those at homes rented for overnight occupancy, are exempt from both regulations.

### Implications for Public Health Practice

Health department officials who perform waterborne disease investigations should prioritize testing any identified hot tub as a potential source of exposure when Legionnaires disease cases meet CDC’s outbreak definition (*9*). Public health officials should conduct on-site field visits, collect and test samples, and use all available tools, including public nuisance laws if needed, to protect public health. Regular postremediation follow-up samples should be collected to ensure long-term control. Vacation and other short-term rental property owners should be informed of the risks associated with improperly managed hot tubs, particularly for persons at increased risk for severe illness, including those with underlying medical conditions.

## Footnotes

* Interviews revealed that five additional guests stayed at the rental property and might have used the hot tub. These additional guests did not report any symptoms and therefore were not interviewed or part of any investigative process. Patients C, D, and E were investigated as a separate cluster. No additional exposures sources were identified.

** The NYSDOH program Protect Yourself from Legionnaires Disease at Home guidance was updated to include additional guidance for hot tub and rental property owners. CDC also offers guidance for vacation rental owners. Legionnaires Disease Prevention | Providing a Home for Guests, not *Legionella* | CDC

†† Facilities include buildings of general hospitals that provide inpatient services or buildings of residential health care facilities providing a health-related service, such as lodging, board, or physical care.
